# Understory vegetation mediates permafrost active layer dynamics and carbon dioxide fluxes in open-canopy larch forests of northeastern Siberia

**DOI:** 10.1371/journal.pone.0194014

**Published:** 2018-03-22

**Authors:** Michael M. Loranty, Logan T. Berner, Eric D. Taber, Heather Kropp, Susan M. Natali, Heather D. Alexander, Sergey P. Davydov, Nikita S. Zimov

**Affiliations:** 1 Department of Geography, Colgate University, Hamilton, NY United States of America; 2 School of Informatics, Computing, and Cyber Systems, Northern Arizona University, Flagstaff, AZ United States of America; 3 Woods Hole Research Center, Falmouth, MA United States of America; 4 Department of Forestry, Forest and Wildlife Research Center, Mississippi State University, Mississippi State, MS United States of America; 5 Northeast Science Station, Pacific Institute for Geography, Far East Branch, Russian Academy of Sciences, Cherskii, Republic of Sakha, Yakutia, Russia; University of Copenhagen, DENMARK

## Abstract

Arctic ecosystems are characterized by a broad range of plant functional types that are highly heterogeneous at small (~1–2 m) spatial scales. Climatic changes can impact vegetation distribution directly, and also indirectly via impacts on disturbance regimes. Consequent changes in vegetation structure and function have implications for surface energy dynamics that may alter permafrost thermal dynamics, and are therefore of interest in the context of permafrost related climate feedbacks. In this study we examine small-scale heterogeneity in soil thermal properties and ecosystem carbon and water fluxes associated with varying understory vegetation in open-canopy larch forests in northeastern Siberia. We found that lichen mats comprise 16% of understory vegetation cover on average in open canopy larch forests, and lichen abundance was inversely related to canopy cover. Relative to adjacent areas dominated by shrubs and moss, lichen mats had 2–3 times deeper permafrost thaw depths and surface soils warmer by 1–2°C in summer and less than 1°C in autumn. Despite deeper thaw depths, ecosystem respiration did not differ across vegetation types, indicating that autotrophic respiration likely dominates areas with shrubs and moss. Summertime net ecosystem exchange of CO_2_ was negative (i.e. net uptake) in areas with high shrub cover, while positive (i.e. net loss) in lichen mats and areas with less shrub cover. Our results highlight relationships between vegetation and soil thermal dynamics in permafrost ecosystems, and underscore the necessity of considering both vegetation and permafrost dynamics in shaping carbon cycling in permafrost ecosystems.

## 1. Introduction

Widespread observations of increasing permafrost (perennially frozen ground) temperatures throughout the northern hemisphere in recent decades [1] indicate that large-scale permafrost thaw is likely underway and poised to continue with climate warming. Increased permafrost thaw is important for a number of reasons. Among the most prominent is the large amount of organic carbon stored in permafrost [2] that will become vulnerable to decomposition and transfer to the atmosphere as greenhouse gasses when thawed [3], constituting a potentially large climate feedback [4]. Permafrost thaw may also alter local hydrology and nutrient availability, leading to changes in vegetation composition [5,6] that will result in multiple climate feedbacks related to altered land surface albedo and evapotranspiration [7].

In permafrost soils, the depth of the seasonally thawed active layer is often used as a diagnostic measure of permafrost status. Annual thaw depth measurements can be used to monitor permafrost responses to temporal variation in climate [8,9] while spatially distributed measurements can help to elucidate processes that underlie climate responses at scales ranging from ecosystems to continents [10–12]. In addition to serving as an indicator of permafrost status, thaw depth measurements characterize the portion of the soil column that is available for biological activity, including root growth, plant acquisition of water and nutrients, and decomposition of organic matter. Deepening of the active layer is often associated with enhanced ecosystem respiration (R_ECO_ [13]). Understanding both drivers and consequences of active layer dynamics and how they co-vary within and between ecosystems is critical for predicting carbon cycle responses to continued climate warming in permafrost ecosystems.

In the simplest terms, active layer warming and permafrost thaw is driven by ground heat flux, which is governed by ecosystem influences on surface energy partitioning [14] and soil properties that influence heat transfer [15]. At local scales, surface energy exchange may be influenced by the effects of vegetation cover on ground temperature and moisture via radiation interception [16] and partitioning of sensible and latent heat fluxes [17–20], the insulating effects of snow cover [21], and changes in thermal conductivity associated with soil water content [22]. A potential consequence of such variability is persistent subsurface heterogeneity in active layer depths that, in addition to controlling the amount of unfrozen carbon, may also influence R_ECO_ via impacts on the distribution of heat and water within the soil column [23].

In this study, we examine spatial variability in active layer depth and ecosystem carbon fluxes associated with understory vegetation composition and biomass in an open-canopy larch forest in northeastern Siberia. Larch forests comprise a large portion of the continuous permafrost zone and have low canopy cover [24], meaning that understory vegetation plays a crucial role in regional permafrost and carbon dynamics [25]. Specifically, we address two research questions: 1) do thaw depth and soil temperature vary between common understory vegetation types? and 2) does ecosystem respiration differ among key vegetation types, and if so are these differences related to active layer depth?

## 2. Methods

### 2.1 Study site

**Fig 1:**
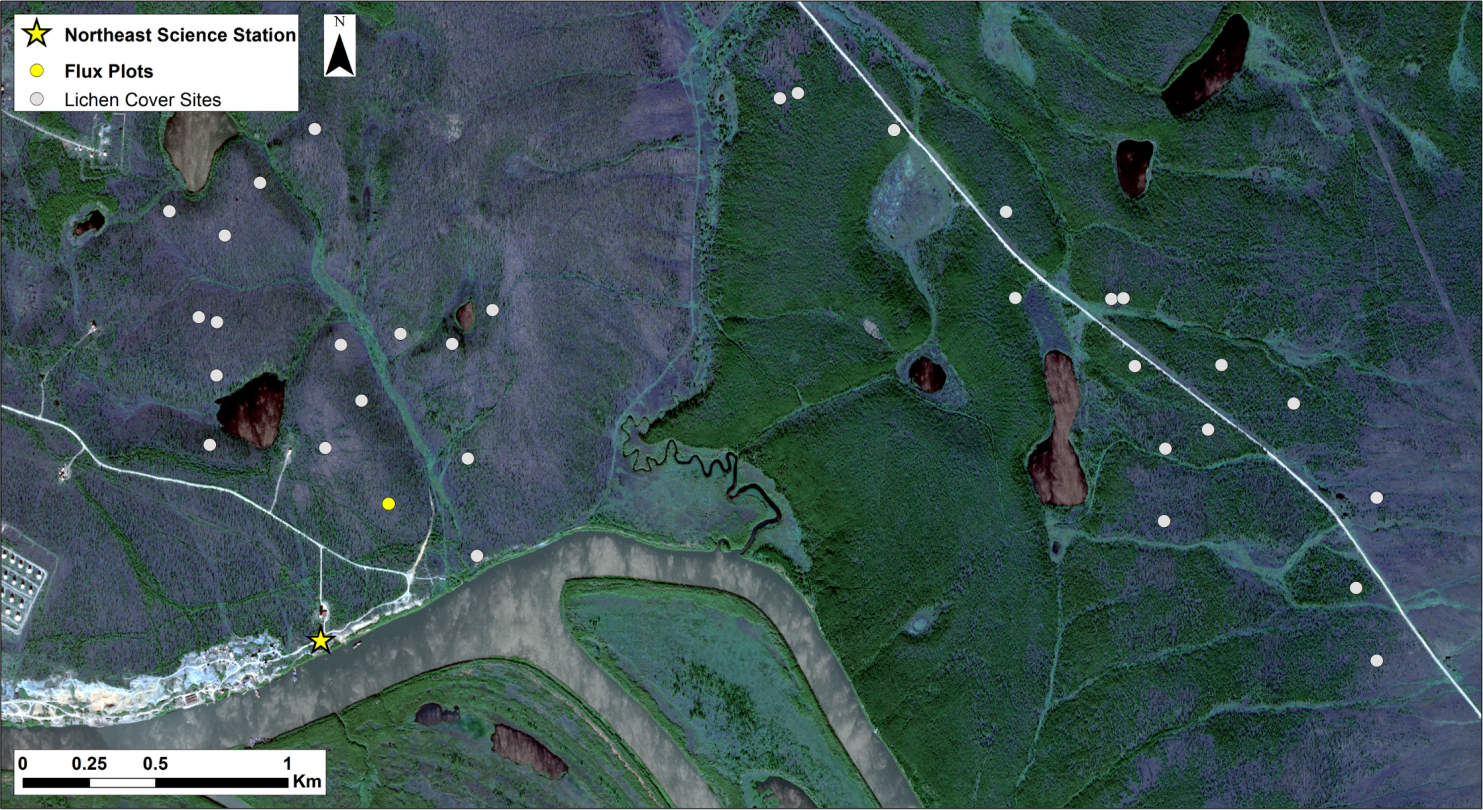
Map of the study area showing the Northeast Science Station and measurement locations. The yellow circle denotes the flux and soil temperature timeseries measurement site, and gray points indicate locations of lichen and canopy cover measurements. Sites in the western half of the map, including the flux plots, are in the Y4 watershed while those along the road in the eastern half are in the Y3 watershed. Stands in the Y3 watershed are approximately 50 years old and of relatively high-density, while the stands in Y4 are older (~150 years) and have generally lower forests density. Background image is a WorldView2 true color composite from 21 August 2012 provided by the Polar Geospatial Center.

Our study was conducted in two small watersheds (Y3 & Y4 ~20km^2^; Fig 1) [26] at the Northeast Science Station (NESS) near the town of Cherskii (68°47’N, 161°20’E) along the Kolyma River in the Sakha (Yakutia) Republic of Russia on private land with permissions arranged via NESS. For the 1986–2015 period the mean annual temperature was -10°C, with mean January and July temperatures of -32°C and 13°C, respectively and average annual precipitation is 218 mm with 85 mm occurring as rain, and 133 mm as snow [27]. Mean summer temperatures increased ~1°C from 1938 to 2009, though there was no systematic change in annual precipitation over this period [28].

Forests in the area are comprised entirely of larch (*Larix cajanderi*). Understory vegetation includes deciduous (*Betula nana exilis*, *Betula nana divartica*, *Salix* spp, *Alnus fruticosa*) and evergreen (*Vaccinium vitis idaea*, *Empetrum nigrum*, and *Ledum decumbens*) shrubs, mosses (including *Aulacomnium turgidum* and *Polytrichum* spp), and lichens (including *Cladonia* rangiferina, *Cetraria cuculata*, and *Stereocaulon tomentosum*). Larch density is a function of fire history and ranges from 0.05 to ~4 tree m^-2^ across early to late successional stands (20–200 years; [29]). For this study, we characterized differences between lichen mats and adjacent shrub-moss patches and measured the distribution of lichen mats in stands throughout the Y3 and Y4 watershed. Here we note that the spatial distribution of vegetation can be strongly influenced by microtpography related to geomorphic dynamics [30] or permafrost dynamics such as ice-wedge polygons [31]. This does not appear to be the case for our study sties because there are virtually no vertical differences between lichen mats and adjacent shrub-moss patches in our study sites. Instead, it is likely that the distribution of understory vegetation, and specifically lichen mats are a function of interactive influences of overstory forest cover, variability in post-fire soil conditions, and species interactions. In this study we examine how understory vegetation varies, and what conditions vary with vegetation, rather than seeking to determine specific causal mechanisms.

### 2.2 Data collection

To understand the distribution of understory vegetation across the Y3 and Y4 watersheds, we measured percent cover of understory vegetation along with larch canopy cover in 35 stands (Fig 1). At each stand, we established three parallel 20 m long transects spaced 8–10 m apart. At the end of each transect, we visually estimated understory percent cover in 1 m^2^ plots (6 per stand). The plots were divided into four quadrats and in each we estimated deciduous and evergreen shrub, forb, graminoid, moss, lichen, and non-vegetated percent cover. Time constraints prevented us from using the point intercept method, which can be more accurate [32]. At the center of each transect, we measured canopy cover using a hemispherical densiometer.

Within one low-density (0.06 ± 0.03 trees m^-2^) late succession (~178 year old) larch stand in the Y4 watershed we examined relationships between understory vegetation cover, permafrost thaw depth (TD), ecosystem thermal characteristics, and fluxes of CO_2_ and H_2_O. At this particular stand, understory vegetation cover is generally dominated by *Betula nana middendorffi*, atop a moss layer, interspersed with mats of *Cladonia* spp. ranging from 0.25–2.0 m^2^ in size. To examine seasonal differences in soil temperature at this stand, we installed HOBO Pendant temperature sensors (Onset Corp, Bourne, MA) at a depth of 10 cm in the soil beneath 0.25 m^2^ lichen mats (n = 9) and shrub/moss patches (n = 9). The sensors recorded hourly soil temperature (T_soil_) from July 2012 until June 2014. Here we also collected soil samples from lichen mats (n = 6) and shrub/moss patches (n = 6) in order to characterize soil properties. We used a soil saw to collect 10cm by 10cm to a depth of approximately 20cm. For each sample we recorded the depth of the organic horizon. Subsamples from the organic and mineral horizons were oven dried at 60°C for 48 hours to determine gravimetric soil moisture, and then placed in a muffle furnace at 450°C for 5 hours to quantify organic matter content using the loss-on-ignition method.

In summer 2013, we measured CO_2_ and H_2_O fluxes and thaw depth at separate 0.25 m^2^ plots dominated by lichen (L; n = 5), low-density shrubs with moss understory (SM; n = 5), and high-density shrubs with little moss in the understory (S; n = 5). These flux plots were located approximately 20m away from those instrumented for T_soil_ measurements and where the soil samples were collected. A total of 174 flux measurements (87 light and dark) were made at these plots between July 17 and August 5, 2013 on seven days with low wind and no rain. On each day we randomly selected the order in which plots were measured. At each plot, we measured CO_2_ and H_2_O fluxes using a LI-COR 840 infrared gas analyzer (IRGA; LI-COR Biosciences, Lincoln, NE) with a manual closed chamber system. A transparent acrylic chamber (50x50x50 cm) was used to measure evapotranspiration (ET) and net ecosystem exchange of CO_2_ (NEE), and an opaque cover was used to measure fluxes under dark conditions (i.e., R_ECO_). A plastic skirt and chain were used to establish an airtight seal between the chamber and the ground. Concentrations of CO_2_ and H_2_O were recorded every second for approximately two minutes with a tablet connected to the IRGA. To calculate gas fluxes, we fit a slope to each set of concentration measurements, using only the linear portion of curve, omitting data without significant linear relationships [33].

At each plot, with each set of fluxes, we also measured soil temperature from 0-5cm depth (T_soil_) and air temperature (T_air_) using a thermocouple (Fisher Scientific, Waltham, MA), photosynthetically active radiation (QSO-PAR Decagon Devices, Pullman, WA), surface soil moisture (GS-3 Decagon Devices, Pullman, WA), and radiometric surface temperature (T_surf_; Appogee Instruments, Logan, UT). We also measured TD on the north and south sides of each plot by inserting a graduated metal rod into the ground until firm resistance was met. Twice at the beginning of the field season we measured soil thermal conductivity (K_S_) integrated over 0–5 cm depth (KD-2, Decagon Devices, Pullman, WA). Half hourly values of T_air_ and air pressure logged to a HOBO Microstation (Onset Corp, Bourne, MA) were matched to the corresponding fluxes according to time of measurement. T_air_ from the meteorological station were used to fill chamber-level gaps due to instrument failure. On three occasions, we also measured the normalized difference vegetation index (NDVI) for each plot (SRS-Nr, Decagon Devices, Pullman, WA). At each flux plot, we measured the basal diameter for Betula spp. and Salix spp. shrubs and then calculated aboveground biomass using regional allometric equations [34]. We also visually estimated percent cover of the dominant plant functional types.

At the same low-density stand where fluxes were measured we quantified subsurface heterogeneity related to surface vegetation using electrical resistivity imaging [35,36]. We measured ERI along ~10m linear transects centered on lichen mats approximately 1–2 m in diameter. ERI is a minimally invasive technique applied by injecting a direct current into the ground and measuring differences in potential along a two-dimensional electrode array. The result of a resistivity survey is a series of point measures that depend upon electrode geometry and an assumption of subsurface homogeneity. An inversion model was used to calculate true resistivity values for the heterogeneous subsurface and generate resistivity images. Roll-along resistivity surveys were conducted using a Syscal Kid resistivity meter (Iris Instruments, Orleans, France) with 24 electrodes spaced 20 cm apart. The 30cm graphite electrodes were inserted in the ground at 45-degree angles in order to maximize electrode contact and minimize vertical distortion, and data were sampled using a Schlumberger sampling array. At 20cm intervals along each ~10 m long transect, we measured TD with a graduated metal rod.

All resistivity surveys were inverted using Res2DInv software [37]. The high resistivity values and contrasts present in permafrost ecosystems introduce challenges with respect to data inversion [38]. In order to avoid over-fitting that leads to extremely high resistivity values [39], we limited the number of model iterations to five. We used a robust inversion because it more adequately captures sharp gradients characteristic of permafrost ecosystems [35]. Additionally, prior research shows resistivity inversions in permafrost ecosystems to be highly sensitive to a model smoothness parameter (λ) [38] that controls the degree to which the model is constrained by individual data points. We used the default initial value of 0.15 in Res2Dinv, and then confirmed our results by generating additional inversions with higher λ values in order to confirm that modeled features were real and not inversion artifacts [40]. The resistivity inversion uses the finite element method for forward modeling, where the surface of interest is discretized into a series of cells, and we varied the number of cell divisions between points as well.

All data analyses were performed in R version 3.2.3 [41]. Preliminary data analyses were conducted to ensure that all data satisfied assumptions of normality associated with each statistical test. For time series T_soil_ and soils data collected beneath lichen mats and shrub/moss patches we tested for differences in soil characteristics among vegetation types using two-sample t-tests. We used a one-way Analysis of Variance (ANOVA) with vegetation type as the fixed effect to examine differences in biological and physical variables related to surface energy partitioning, and also to carbon dynamics measured at the flux plots. A post-hoc Tukey’s Honest Significant Difference test was used to determine whether differences between each vegetation type were significant. Ordinary least squared regressions were performed across all days to examine variability in R_ECO_ responses to soil and air temperature across each surface cover type (e.g. shrub, lichen, and shrub-lichen). All data and code used for analyses are freely available at the following url https://github.com/mloranty/lichen_pft/tree/v2.0 [33].

## 3. Results

### 3.1 Vegetation distribution and active layer properties

**Fig 2:**
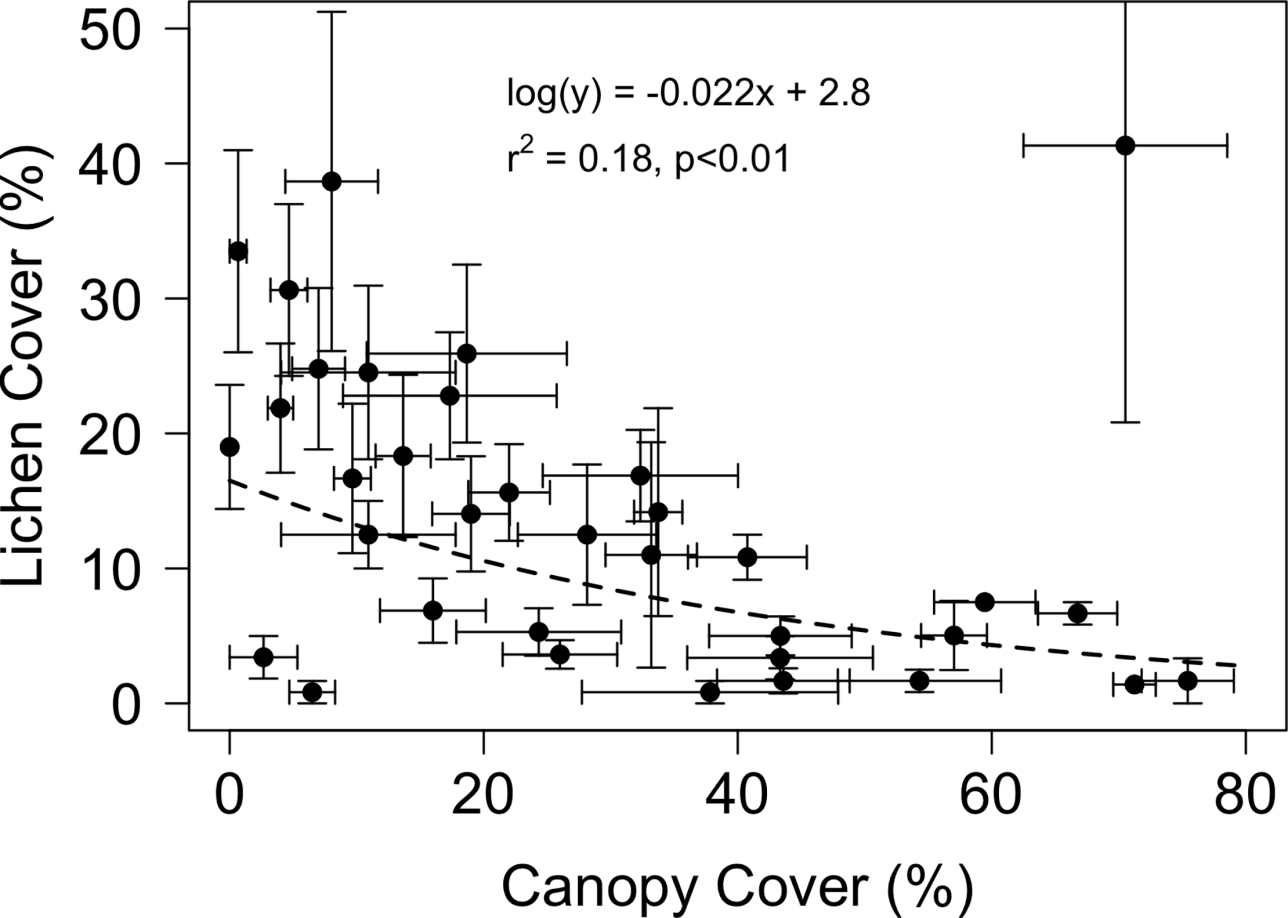
Relationship between larch canopy cover and lichen percent cover. Areal percent cover of lichen declined non-linearly with increasing larch canopy cover for 35 stands in the study area. Lichen cover was estimated visaually and larch canopy cover was measured with a hemispherical densitometer.

**Fig 3:**
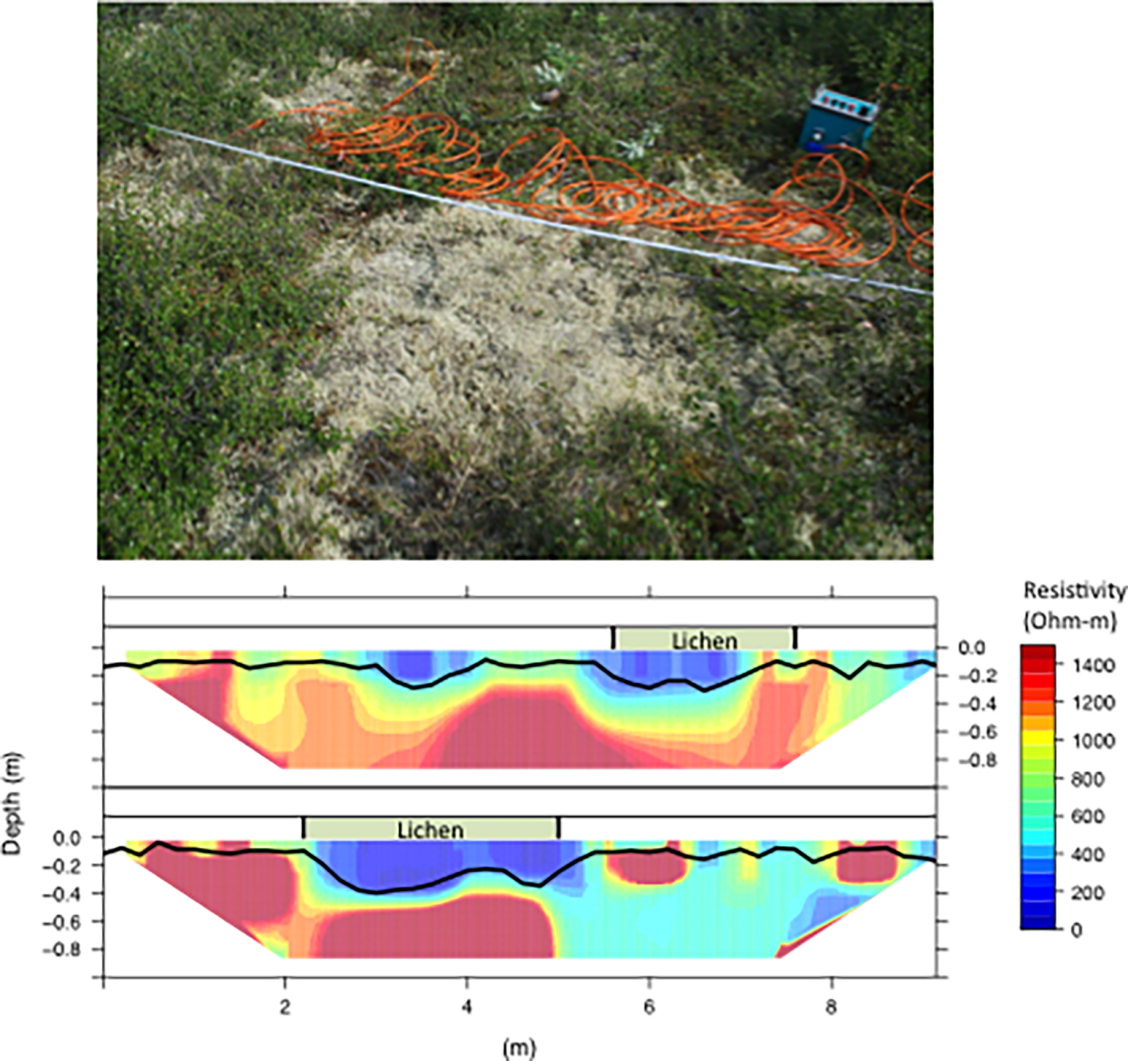
Surface and subsurface characteristics of understory lichen mats. Photograph showing lichen mat and resistivity survey near Cherskii, Siberia. Two representative resistivity profiles where colors indicate resistivity, black lines represent thaw depth measurements, and green boxes labeled *lichen* above each profile denote the location of lichen mats. Note the deep areas of low resistivity (blue) and thaw depth beneath lichen.

Lichen occurred widely in the study area and had notable impacts on resistivity, thaw depth, and soil temperature. Areal percent cover of lichen mats was 16.5 ± 2.5% (±1 SD) with a range of 3–33% across the 35 forest stands sampled in this watersheds. Mean percent cover of deciduous shrubs and moss were (37.6 ± 2.9% and 39.2 ± 4.1%, respectively). The distribution of lichen mats was not uniform, but rather exhibited a significant (p < 0.05) inverse relationship with larch canopy cover (Fig 2). The ERI tomography and thaw depth measurements in mid-July revealed low resistivity beneath lichen mats relative to adjacent areas dominated by shrubs and moss, indicative of deeper thaw and/or saturated conditions beneath lichen mats (Fig 3). Concurrent measurements of thaw depth aligned well with resistivity patterns, showing deeper thaw beneath lichen mats (Fig 3).

**Fig 4:**
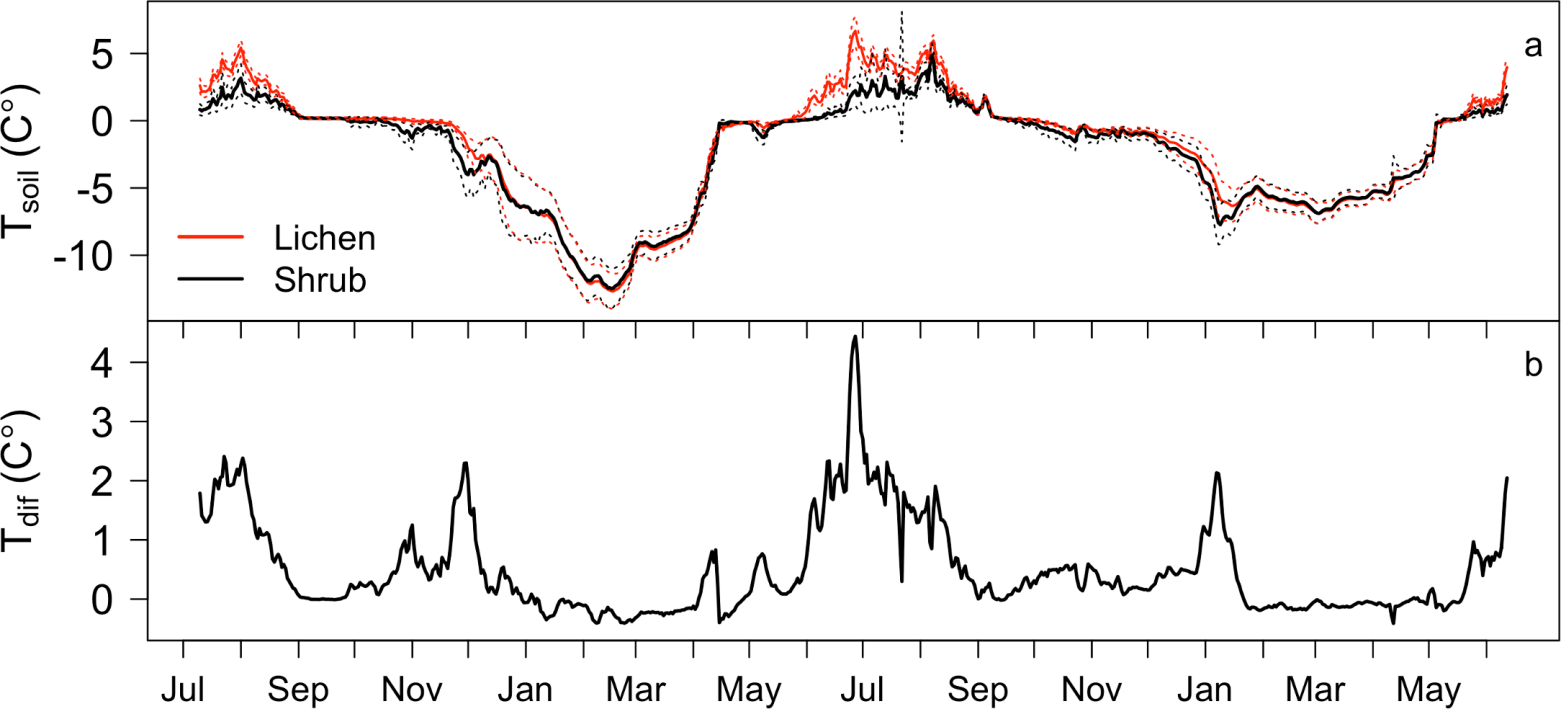
Soil temperatures beneath lichen and shrub understory vegetation. (A) Time series of daily mean T_soil_ at 10 cm depth beneath lichen mats (n = 9) and shrub patches (n = 9) from July 2012 –June 2014. Dotted lines indicate one standard deviation. (B) The difference between daily mean lichen and shrub temperatures (lichen-shrub).

At the intensively measured low-density stand soil temperature measured at 10 cm depth from July 2012 –June 2014 revealed elevated temperatures beneath lichen mats relative to adjacent vegetation patches dominated by shrubs and mosses (Fig 4). These differences were largest during the early to mid growing season (~2°C), and gradually decreased towards the end of the growing season. Differences were minimal at the onset of fall freeze-back, but then increased again as soils beneath lichen mats spent longer time in the ‘zero curtain’ period, after which differences were minimal during the winter. Aggregated seasonal temperatures (Table 1) were significantly different during the growing season (June-August; p < 0.01) and autumn (September–November), but not during winter (December-February) or spring (March-May).

**Table 1:** Seasonal soil temperatures at 10cm depth beneath lichen mats and shrub moss patches^1^.

Season	T_soil_ (°C)	
	Lichen^2^	Shrub/Moss
2012 SON**	0.33 (0.11)	0.01 (0.23)
2013 DJF	-6.34 (1.46)	-6.62 (1.56)
2013 MAM	-5.61 (0.49)	-5.58 (0.59)
2013 JJA**	3.17 (0.48)	1.68 (0.47)
2013 SON*	0.15 (0.13)	-0.16 (0.28)
2014 DJF	-3.57 (0.85)	-3.96 (0.85)
2014 MAM	-4.61 (0.52)	-4.52 (0.53)

^1^ Significant differences at

* = p<0.05

** = p<0.01

^2^ Lichen n = 9; Shrub/Moss n = 9

The soil organic layer was twice as thick (Table 2; p = 0.00001) beneath shrub/moss plots (12.3 ± 1.5 cm) in comparison to lichen mats (6.0 ±1.4 cm). Gravimetric soil moisture and soil organic matter content in the organic soil horizon were significantly higher beneath shrub/moss patches relative to lichen mats (Table 2; p = 0.00008 and p = 0.0002 respectively); however, soil moisture and organic matter content within the mineral soil did not differ significantly between vegetation types (Table 2).

**Table 2:** Soil properties for lichen mats and shrub/moss patches in a low-density larch stand in Northeastern Siberia^1^.

		Organic Soil Layer		Mineral Soil Layer	
Vegetation	Organic Depth	Soil Moisture	SOM	Soil Moisture	SOM
Type	(cm)	(%)	(%)	(%)	(%)
Lichen^2^	6.0 (1.4)**	52.7 (5)**	39.0 (6.9)**	36.7 (6.6)	13.8 (4.6)
Shrub/Moss	12.3 (1.5)**	70.9 (3.6)**	72.5 (10.7)**	39.9 (10.2)	17.0 (6.1)

^1^ Significant differences at

* = p<0.05

** = p<0.01

^2^ Lichen n = 6; Shrub/Moss n = 6

### 3.2 Vegetation influences on carbon and water dynamics

Plots utilized for flux measurements exhibited clear differences in biological and physical characteristics among vegetation types (Table 3). Shrub plots had an average of 210 ± 46 g shrub aboveground biomass, which was significantly higher than the 69 ± 15 g in shrub-moss plots, while only one lichen mat had a shrub (0.9 ± 0.9 g). Percent moss cover did not differ significantly between shrub-moss plots (52 ± 11%) and shrub plots (34 ± 9%). Despite significant differences in shrub biomass, NDVI did not differ significantly between shrub-moss (0.61 ± 0.01) and shrub plots (0.63 ± 0.01), though both were significantly higher (ANOVA, p < 0.05) than lichen mats (0.40 ± 0.01). Thermal conductivity was significantly higher (ANOVA, p < 0.05) in lichen mats (0.40 ± 0.06 W m^-1^ K^-1^) in comparison to shrub-moss (0.12 ± 0.02 W m^-1^ K^-1^) and shrub plots (0.07 ± 0.01 W m^-1^ K^-1^). Thaw depth below lichen mats was significantly higher (ANOVA, p < 0.05) at the beginning (July 19; 72 ± 4 cm) and end (Aug 3; 79 ± 3 cm) of the study period than either shrub-moss (26 ± 4 cm and 36 ± 6 cm) or shrub plots (34 ± 9 cm and 48 ± 8 cm). Differences in thermal conductivity and thaw depth between shrub-moss and shrub plots were not statistically significant.

**Table 3:** Summary of key vegetation and physical parameters for CO_2_ and H_2_O flux plots measured between 17 July and 5 August 2013^1^.

Plot Type	Shrub Biomass	Moss Cover^2^	NDVI^3^	K_S_	TD—July 19	TD—Aug 3
	(g)	(%)		(W m^-1^ K^-1^)	(cm)	(cm)
Lichen^4^	0.9 (0.9)^a^	0 (0)^a^	0.40 (0.01)^a^	0.40 (0.06)^a^	71.9 (4.2)^a^	78.5 (3.4)^a^
Shrub-Moss	69.1 (14.7)^a^	52 (11)^b^	0.61 (0.01)^b^	0.12 (0.02)^b^	26.4 (4.0)^b^	36.2 (6.1)^b^
Shrub	210.6 (46.0)^b^	34 (9)^b^	0.63 (0.01)^b^	0.07 (0.01)^b^	33.7 (8.7)^b^	48.1 (7.9)^b^

^1^Letters indicate significant differences in measured variables between plot type

^2^Understory precent cover estimate includes moss cover beneath shrub canopies.

^3^Means are an average of three observations taken during the study, no temporal patterns were observed

^4^Lichen n = 5; Shrub-Moss n = 5; Shrub n = 5

Examination of variables related to surface energy partitioning may help elucidate drivers of observed differences in TD. To accomplish this, we present plot-level differences in T_soil_, T_air_, T_surf_, PAR, and ET (latent heat flux) for two days with contrasting environmental conditions (Table 4). On 1 August 2013 all fifteen plots were sampled between 16:00 and 18:00 when temperature was moderate and PAR was low, and both were invariant across the sample period. During this time, the difference between T_surf_ and T_air_ (T_dif_) was slightly positive (warmer) on lichen mats while the shrub patches were approximately 1°C cooler than the air, but differences in T_surf_ and T_dif_ between vegetation type were not statistically significant. On 3 August 2013 all plots were sampled between 11:30 and 13:30 when T_air_ and PAR were relatively high compared to 1 August. On this occasion, T_surf_ was substantially higher than T_air_ across all vegetation types; with lichen T_surf_ being on average 12.0 ± 2.6°C warmer than T_air_ whereas T_surf_ was 6.3 ± 1.5°C and 4.8 ± 1.6°C warmer than T_air_ for shrub-moss and shrubs, respectively (Table 4). Differences in ET between vegetation types were not significantly different on either day. However, on August 3 when vegetation surfaces were substantially warmer than the air, lichen had the lowest ET and the highest T_surf,_ while on August 1 there were no appreciable patterns in ET and small differences in T_surf_. On both days there were significant differences in T_soil_ (ANOVA, p < 0.05) that were consistent with observed patterns of TD and time series observations of T_soil_ at the site.

**Table 4:** Variability in soil, air, and radiometric surface temperatures, photosynthetically active radiation, and evapotranspiration between vegetation types measured at flux-plots for two sampling periods with contrasting meteorlogical conditions during the 2013 field season^1^.

		T_soil_	T_air_	T_surf_	PAR^2^	Evapotranspiration
		(°C)	(°C)	(°C)	(μmol m^-2^ sec^-1^)	(mmol H_2_O m^-2^ sec^-1^)
1 August	Lichen^3^	4.4 (0.4)^a^	6.6 (0.1)	6.9 (0.8)	248 (27)	0.26 (0.08)
16:00–18:00	Shrub-Moss	2.3 (0.4)^b^	6.6 (0.1)	5.8 (0.8)	248 (27)	0.19 (0.11)
	Shrub	2.8 (0.6)^b^	6.6 (0.1)	5.6 (0.7)	233 (41)	0.20 (0.05)
3 August	Lichen	4.1 (0.3)^a^	8.0 (0.1)	20.0 (2.7)^a^	967 (44)	1.07 (0.31)
11:30–13:30	Shrub-Moss	2.1 (0.3)^b^	8.2 (0.2)	14.5 (1.6)^ab^	1062 (74)	1.73 (0.32)
	Shrub	2.9 (0.4)^b^	8.2 (0.2)	13.0 (1.8)^b^	1062 (74)	1.67 (0.38)

^1^Letters indicate significant differences in measured variables between vegetation type. Variables without letters have no significant differences at p < 0.05

^2^Photosynthetically Active Radiation.

^3^ For each day Lichen n = 5; Shrub-Moss n = 5; Shrub n = 5

**Fig 5:**
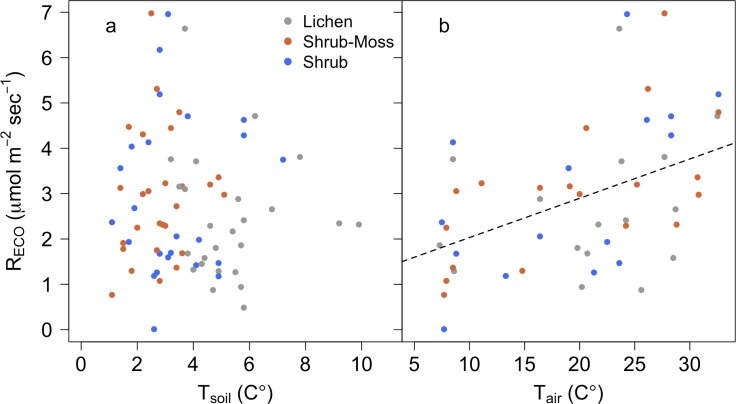
Variation in ecosystem respiration with air and soil temperatures. (A) R_ECO_ was not significantly related to T_soil_. (B) R_ECO_ was was positively related to air temperature when observations were polled across plant communities and sampling periods (intercept = 1.16, slope = 0.09, adj r^2^ = 0.19, p < 0.01).

Deeper thaw depths and warmer soil temperatures associated with lichen did not lead to higher R_ECO_ (Table 5). Across the study period, average R_ECO_ was not significantly different across vegetation types; 2.42±0.27 μmol m^-2^ sec^-1^, 2.92±0.27 μmol m^-2^ sec^-1^, and 2.91±0.36 μmol m^-2^ sec^-1^ for lichen, shrub-moss, and shrub plots, respectively. Average NEE across the study period was negative in shrub plots (-0.54±0.67 μmol m^-2^ sec^-1^), indicating net carbon uptake. This was significantly lower than NEE values of 2.01±0.58 μmol m^-2^ sec^-1^ and 2.46±0.30 μmol m^-2^ sec^-1^ in shrub-moss and lichen plots, respectively (ANOVA; p < 0.05). Individual measurements of R_ECO_ were linearly related to air temperature (adj r^2^ = 0.19, p < 0.01) but not 0–5 cm soil temperature (Fig 5). We did not observe significant relationships between R_ECO_ and air or soil temperatures within individual vegetation types.

**Table 5:** Comparison of mean values of key carbon and water fluxes, and key meteorological drivers for each vegetation type averaged across the study period between 17 July and 5 August 2013^1^.

Vegetation	T_soil_	T_air_	T_surf_	T_dif_	PAR^2^	NEE^3^	R_ECO_	Evapotranspiration
Type	(°C)	(°C)	(°C)	(°C)	(μmol m^-2^ sec^-1^)	(μmol CO_2_ m^-2^ sec^-1^)	(μmol CO_2_ m^-2^ sec^-1^)	(mmol H_2_O m^-2^ sec^-1^)
Lichen^4^	5.2 (0.3)^a^	16.8 (1.3)	13.0 (0.9)	3.8 (1.0)^a^	786 (65)	2.46 (0.30)^a^	2.42 (0.27)	0.86 (0.14)
Shrub-Moss	2.9 (0.2)^b^	14.1 (1.0)	13.0 (0.9)	1.1 (0.6)^b^	773 (63)	2.01 (0.58)^a^	2.92 (0.27)	1.16 (0.19)
Shrub	3.4 (0.3)^b^	13.7 (1.1)	12.9 (0.9)	0.8 (0.6)^b^	756 (65)	-0.54 (0.67)^b^	2.91 (0.36)	1.22 (0.16)

^1^Letters indicate significant differences in measured variables between vegetation type at p < 0.05. Variables without letters have no significant differences.

^2^Photosynthetically Active Radiation

^3^Net Ecosystem Exchange of CO_2_.

^4^Lichen n = 29; Shrub-Moss n = 29; Shrub n = 29

## 4. Discussion

### 4.1 Vegetation and active layer dynamics

Our results illustrate the influence of understory plant functional type on permafrost thaw depth, likely due to differences in surface energy partitioning. Specifically, we found that thaw depths were 2–3 times greater under lichen mats than under shrubs or mosses. Low moisture content and limited evapotranspiration from lichens prevent dissipation of latent heat [18], potentially allowing for deeper thaw. On the other hand mosses have a low thermal conductivity and high insulating capacity when dry, due to a high air volume [42,43]. Mosses can thus prevent thawing of permafrost by reducing the transfer of solar heat into the soil [20,44,45]. When damp, mosses have high rates of evapotranspiration that minimize sensible and ground heat fluxes, thus buffering against temperature variations [22] and reducing soil temperature [20,46]. Similarly, a combination of ground shading and relatively high latent heat fluxes may minimize permafrost thaw beneath shrub canopies [16]. The absence of a root system in mosses and lichens limits soil water loss, and may lead to the development of saturated soil [44]. A study by Stoy et al [23] found higher soil temperatures (1 cm and 5 cm depth) beneath lichen and feather moss relative to nearby sphagnum, however, modeled ground heat flux for lichen was relatively low in comparison to mosses. Our study is broadly inline with these previous results; we found that lichen mats had both thinner organic layers with less organic matter and correspondingly higher thermal conductivity, and also higher surface temperatures associated with lower latent heat dissipation. Both of these sets of factors are likely to contribute to warmer soils and deeper active layers beneath lichen mats.

It is also important to consider that variability in micortopography and soil moisture can also influence vegetation distribution [30,31,47]. This is commonly observed in areas with ice-wedge poloygons, where permafrost dynamics create micortopography with low wet areas adjacent to higher drier areas. Elevation differences in these cases are typically on the order of 10s of cm and can be the dominant controls on moisture [48], which in turn impacts vegetation community distribution. This does not seem to be the case at our sites as there is no microtopography of this nature. However it is still possible that, in addition to overstory forest cover, lichen distribution is partially determined by post-fire soil conditions that have persisted to the present. In this case the differences in thermal properties that we observed may actually reinforce differences in active layer properties partially responsible for vegetation distribution. Fire typically alters active layer depths for up to fifty years post-fire [49,50], and ecosystem recovery typically promotes recovery of the permafrost table [51,52]. In our study sites the high-density stands are among the youngest (~50yrs), whereas the lower density sites with higher lichen abundance are upwards of 150 years old, indicating that our estimates of active layer heterogeneity are not likely the results of fire legacy effects. In any case, improved understanding of active layer heterogeneity in Siberian larch forests requires comprehensive patch-scale observations of understory vegetation.

### 4.2 Thaw depth and ecosystem respiration

Previous studies suggest that increased thaw depth can lead to greater carbon loss through increased heterotrophic respiration if soil carbon and quality with depth [17–20,53–55]; however, we found no relationship between thaw depth and R_ECO_. Autotrophic respiration can comprise a substantial portion of R_ECO_ in permafrost ecosystems [13]. It is likely that higher autotrophic respiration compensated for lower heterotrophic respiration in shrub plots, which had higher aboveground biomass and shallower permafrost thaw depths than other vegetation types. The observed dependence of R_ECO_ on air temperature rather than soil temperature supports this idea. Additionally, differences in the quantity and quality of soil carbon beneath different vegetation types may also contribute to variability in R_ECO_, and our observations of thicker organic soil layers with higher organic matter content suggest soil carbon differences may have been a factor in this study. For example, the low biomass, slow turnover and low litter input of lichens could limit the amount of soil carbon available for decomposition in lichen patches relative to areas dominated by mosses or shrubs [56]. Thus, decreased soil organic matter may negate carbon flux from increased thaw depth. Conversely, high soil organic matter content of soil beneath shrub patches may enhance R_ECO_ from moderate thaw depths. Alternatively, labile carbon may have already decomposed beneath lichen patches with deeper thaw depths. Spatial variation in the amount and lability of soil C are also likely influenced by fire, which is the dominant disturbance in the region [29,57].

Soil moisture may also influence relationships between thaw depth and R_ECO_ via moisture limitations on decomposition [58–60] that can be regulated by vegetation [44,58,61]. Several studies in Alaska have identified accumulation of water at the base of the active layer as a potential explanation for lack of observed relationships between thaw depth and R_ECO_ [21,53], and this could be plausible at our study site as well. Similarly, Zona et al [62] observed higher R_ECO_ at micro sites with shallower thaw depth and lower water tables. In these cases, suppression of R_ECO_ by soil moisture may be offset by CH_4_ efflux associated with anaerobic respiration [63,64]. Our observations of delayed autumn freeze-back suggest a higher amount of soil moisture beneath lichen mats relative to adjacent areas dominated by shrubs and mosses; however, this does not necessarily correspond to saturated soil at the base of the active layer and could also result simply from deeper thaw depths. Other studies have observed spatial variability in CH_4_ across different vegetation types [65], but it is unclear if CH_4_ efflux varies with thaw depth at our study site. Our measurements of R_ECO_ are snapshots taken during the peak of the growing season; however, non-growing season fluxes often determine sign and magnitude of the annual carbon balance for an ecosystem [53,66]. Thus year round measurements of CO_2_ and CH_4_ are required to understand the effects of vegetation-mediated active layer dynamics on the understory carbon fluxes in Siberian larch forests.

### 4.3 Implications for ecosystem change

The relationship between plant functional type and thaw depth supports the assertion that vegetation-mediated variability in surface energy partitioning may alter soil thermal and biogeochemical dynamics in permafrost ecosystems [23]. Lichen constitutes upwards of 16% of understory aboveground biomass in low-density larch stands in northeastern Siberia [29] and 8–32% of aboveground biomass among a network of tundra sites in western Siberia [67]. Accounting for this variability will be necessary for accurate estimates of carbon dynamics in these ecosystems. The same is likely to be true for other permafrost ecosystems, assuming the occurrence of similar relationships between thaw depth and plant functional type, particularly where changes in herbivore browsing patterns (e.g. Rangifer tarandus; [68]), fire [69] or replacement by other forms of vegetation [70,71] lead to rapid changes in lichen distribution. In this context, it will be particularly important to determine whether depressed R_ECO_ associated with lichen cover is a function of saturated soils, low rates of heterotrophic respiration, or some combination of both.

At the landscape scale, the type of subsurface heterogeneity that we observed may translate to bidirectional responses of R_ECO_ to interannual climate variability e.g. [72], potentially leading to hotspots for CO_2_ and CH_4_ emissions [64] that vary spatially from year to year. This sort of space-time variation may present challenges in modeling future greenhouse gas emissions from permafrost ecosystems. On the other hand, high-resolution spectral data combined with observed relationships between surface vegetation and thaw depth in these ecosystems may lead to improved understanding of ecosystem-scale variability in active layer dynamics [73–76].

## 5. Conclusions

In open canopy larch forests in northeastern Siberia, variations in understory vegetation over short distances (e.g. < 1 m) correspond to large differences in thaw depth. Our results illustrate a strong interactions between vegetation and active layer dynamics in these ecosystems, where lower latent heat fluxes and higher thermal conductivity in lichen mats lead to deeper thaw depths. These vegetation types have clear spectral differences, and offer the possibility for remote detection of active layer heterogeneity. Despite increased thaw depth beneath lichen mats, we did not observe elevated ecosystem respiration. Here, soil moisture, along with lower soil carbon content may help to explain the absence of elevated ecosystem respiration, relative to adjacent areas with shallower thaw depths. A better understanding of this subsurface variability, particularly thaw depth controls on soil moisture redistribution, will be necessary to accurately quantify the effects of permafrost thaw on ecosystem carbon cycling. To this end, our results indicate the usefulness of electrical resistivity imaging for visualizing active layer heterogeneity. In Siberian larch forests, fire controls on tree density and understory vegetation succession will exert strong controls on variation in active layer and ecosystem carbon dynamics under current and future climates.
